# Physiology of the Liver

**Published:** 1853-01

**Authors:** M. Bernard

**Affiliations:** College of France


Translated for the Stethoscope.

*Physiology of the Liver.*—Lessons of M. Bernard in the College of France.

The organs intermediate between the intestines and the heart are, on the one hand, the venæ portarum, the liver, and the spleen; and on the other hand, the chyliferous vessels, the mesenteric glands, the thoracic duct, and the veins into which it empties. All of these organs evidently modify substances which are absorbed. Galen spoke of these modifications, and insisted on the importance of the liver in regard to them. According to him, it is the function of this organ to decoct the alimentary substances; he compared its action to that which takes place in the formation of wine: one portion was supernatant—this was the bile: another portion descended into the spleen, this was the dregs or splenic sediment; the blood itself was the wine. Galen, ignorant of the circulation, blindly established this theory; yet one may perceive that it has a foundation in truth. It prevailed for fourteen centuries. It was first attacked by Vesalius, and afterwards by Van Helmont, and yet more zealously by Azelli, who first discovered the lymphatic vessels, yet without finding their outlet. Pecquet, lastly, having discovered the reservoir which bears his name,* and the entrance of the chyle into the blood, gave the last blow to the theory of the celebrated Roman physician.

The discovery of the circulation, towards the middle of the

* The dilatation by which the thoracic duct commences [receptaculum chyli] opposite the second lumbar vertebra, is usually called "resevoir ou citerne de Pecquet," by French anatomists. See Cruveilhier, tome 3, p. 141.—Trans.

seventeenth century, completed the entire desertion of the liver in favor of the lungs. Thomas Bartholin, who had participated in the researches of Azelli, sustained with violence the new ideas: he annihilated the functions of the liver; and, as it was of no farther use in the economy, he wrote its *epitaph*. One can hardly form a conception of the passion and stubborn animosity with which these discussions were carried on. We must descend even to the time of Magendie to see the functions of the abdominal venous system restored. It is universally known that this great physiologist has shown, by incontestable experiments, the part which this system plays in the process of absorption.

M. Bernard announces that he inclines to the doctrine of Galen, inasmuch as he will prove the immense importance of the liver. He will advance only upon the basis of rigid experiment, and thereby he hopes to induce his hearers to his mode of thinking. He will not encounter such spirits as those of the professors of Montpellier, before whom Pecquet repeated his experiments. Those professors did not believe it possible to apply to man principles derived from experiments on animals: they indignantly asked, What would become of the science laboriously accumulated, if they suffered it to be thus overturned? Following the example of Riviere, who replied to them that the senses alone should control our belief, it is to the senses that M. Bernard will continually appeal.

It will be necessary to renounce several received ideas derived from collateral sciences when we enter upon questions relative to the process of absorption. Although these sciences have often contributed to the progress of physiology, yet it is certainly an error to suppose that here things take precisely the same course as in a laboratory. If the gastric juice acts in a glass just as it does in the stomach, yet there are other phenomena which depend upon nervous influence, and which cannot be demonstrated by chemistry.

Moreover, dissections and injections cannot show upon the dead subject how certain substances pass from one sanguineous system to another, whereas experiments upon animals exhibit beyond a doubt the changes of course which occur during life. The disagreements of physiologists have frequently no other cause than this, that they experiment under different circumstances.

There are on the surface of the intestines two orders of vessels which absorb, the veins of the portal system and the lymphatics. The first take up all albuminous, nitrogenized, and saccharine soluble matter. The second are loaded chiefly with fatty substances.

M. Blondlot admits that the villosities of the intestinal veins have a peculiar arrangement, by means of which they inhale some substances, such, for example, as the prussiate of potash; while the lymphatic vessels cannot take up this body. This is an error; for M. Bernard has established the fact that the prussiate of potash is found in the chyliferous vessels, only the mesenteric glands, in elaborating the chyle, deprive it of soluble matter, and retain only fatty matters. Sugar also, like the prussiate of potash, is never found in the thoracic duct; but, since its presence has been proved in the primary chyliferous vessels, this is because it is removed by the mesenteric glands.

On the other hand, it is indubitable that many soluble substances are not absorbed. We showed last year, in detailing the curious experiments of M. Bernard, that the poison called *curare veneno*, and the venom of the viper, do not enter the blood, unless there is a lesion of the mucous membrane with which they are in contact; and also that this phenomenon occurs in some other substances: such as pepsin, diastase, and emulsin. The same fact obtains in regard to putrid and virulent matter. If we introduce a putrid substance into the blood, typhoid symptoms intervene; but these do not occur if such a substance only enters the digestive passages—at least, unless these contain sulphuretted hydrogen. As to virulent substances, if they are not absorbed, it is because they happen to be deposited upon a thin layer of mucus, which serves the purpose of epithelium. It is thus that we can explain the experiment of Mr. Cullerier, as well as the singular observation of M. Ricord, both of which are detailed in *L' Union Medicale* of April 23.

When animals are fasting, the vessels of the portal system have an easy task; for then they receive only the blood of the mesenteric arteries. But when digestion commences, alimentary matters, with a flood of secretions besides, by means of absorption, enter the portal veins, which continue moreover to receive in the same quantity the blood of the mesenteric arteries. The liver is then gorged by an enormous mass of liquids; the blood, accumulating in it as in a sponge, greatly augments its volume and deepens its color. When digestion is concluded, the blood resumes its course, and the liver returns to its natural size, form, and color. These phenomena, in fact, are common to all organs which are intermittent in their functions. With the view of determining the amount of blood contained in the liver during the period of digestion, M. Bernard has made the following experiment: He took three rabbits of the same litter, the first was fasting and weighed 650 *grammes*;* its liver weighed 36 grammes; the second, which commenced to digest,

* MEASURES OF WEIGHT.

| French Measures. | Approximate Value |
|---|---|
| 1 centigramme, | 1-5th of a grain. |
| I decigramme, | 2 grains. |
| 1 gramme, | 20 grains. |
| 10 grammes, | 2½ drachms. |
| 100 grammes, | 3 ounces 2 drachms. |
| 1 kilogramme, | 2 pounds. |

and which weighed 690 grammes, presented a liver which weighed 38 grammes; while the third, weighing 700 grammes and in full digestion, had a liver of 60 grammes. Taking into consideration, then, the relative difference in weight, there was still a very notable augmentation of the bulk of the liver during the period of digestion.

We will not follow the professor in the views which he develops in regard to the general arrangement of the circulatory system of the liver, in regard to the ultimate structure of that gland, and in regard to its secretions. It is now perfectly well known, that all animals which possess a liver, are provided with portal veins emptying into that organ, and that the four instances in which the portal vein emptied into the vena cava without traversing the liver, are either contested or are so explained as to prevent them from rendering doubtful the important functions of the liver. We are also acquainted with the different propositions which have been advanced by authors in regard to the origin of the biliary passages: some insisting that they commence by a cul-de-sac—others by the plexuses which surround the hepatic artery and vena porta. As to the products of secretion: one, known from the earliest times under the name of bile, escapes by the intestines; another, the discovery of which is but recent, the sugar, mingling with the blood of the liver, issues from that organ by the hepatic veins and goes to the lungs, where it is destroyed. The blood which arrives at the liver is then decomposed into sugar and bile. May not the bile be in some way the molasses of the sugar? We shall find that there are, moreover, two other products of secretion in the liver.

Faithful to the promise which he made in the beginning, that he would proceed only upon the basis of rigid experiment, M. Bernard concludes these preliminaries by showing his class the method for obtaining chyle and genuine blood of the portal vein Peculiar precautions are necessary in order to analyze comparatively the blood which enters and that which issues from the liver; for there are phenomena in the circulation, unnoticed by authors, which have been the sources of the gravest errors. We have already noticed, in a preceding report (*compte-rendu*) that the current of the blood from the intestines to the liver, ceases as soon as the abdomen is opened, and the compression exercised by the diaphragm and abdominal muscles is removed; the blood from the liver thereupon descends towards the intestines, so that in collecting the contents of the vena porta, we have blood modified by the liver, or at least this blood mingled with that which has not yet entered that organ. This explains the fact that such analyses have not heretofore offered sensible differences.

M. Bernard had a large dog, in full digestion, placed upon his table: he made an incision and placed a ligature upon the trunk of the vena porta; afterwards he killed the animal by puncturing the medulla; he then opened the abdomen; the heart still palpitated. He divided the vena porta below the ligature, and collected nearly an ounce of blood. He next isolated the thoracic duct, which, as well as the other chyliferous vessels, is distinguished by its milk-white color; he cut this before its discharge into the venous system, and procured about two-thirds of an ounce of liquid blood, which was observed to flow more abundantly as the abdominal vessels were compressed. These liquids, thus collected, were reserved for future experiments. In order to fulfill the programme announced at the commencement of the course, we shall divide this report into five paragraphs:

1. Saccharine matters formed in the liver.
2. Fatty matters formed in the liver.
3. Fibrine formed in the liver.
4. The biliary secretions.
5. The circulation of blood in the liver.

